# HIV-1 CCR5 gene therapy will fail unless it is combined with a suicide gene

**DOI:** 10.1038/srep18088

**Published:** 2015-12-17

**Authors:** Aridaman Pandit, Rob J. de Boer

**Affiliations:** 1 Theoretical Biology and Bioinformatics, Utrecht University, Utrecht, 3584CH, The Netherlands

## Abstract

Highly active antiretroviral therapy (ART) has successfully turned Human immunodeficiency virus type 1 (HIV-1) from a deadly pathogen into a manageable chronic infection. ART is a lifelong therapy which is both expensive and toxic, and HIV can become resistant to it. An alternative to lifelong ART is gene therapy that targets the CCR5 co-receptor and creates a population of genetically modified host cells that are less susceptible to viral infection. With generic mathematical models we show that gene therapy that only targets the CCR5 co-receptor fails to suppress HIV-1 (which is in agreement with current data). We predict that the same gene therapy can be markedly improved if it is combined with a suicide gene that is only expressed upon HIV-1 infection.

HIV infects human T cells by binding to the CD4 receptor and the CCR5 or the CXCR4 co-receptor. HIV-1 primarily targets effector/memory (*T*_*EM*_) CD4^+^ T cells because they express CCR5^1^,^2^. Individuals carrying a CCR5 deletion mutation (CCR5Δ-32) are resistant to HIV-1 because HIV-1 does not bind efficiently to the target cells^1^,^3^. Recently, an HIV-1 infected individual, the “Berlin patient”, was cured after receiving a hematopoietic stem cell (HSC) transplant from a donor homozygous for the CCR5Δ-32 mutation^4^. Mathematical, experimental, and clinical studies have demonstrated that targeting the CCR5 co-receptor can reduce the HIV-1 viral load^1^,^4^,^5^.

Current ART therapies suppress HIV-1 and have drastically improved the survival of HIV-1 infected individuals, but must be administered lifelong. Gene therapy therefore offers an attractive alternative to ART. Current gene therapies aim to create a population of CD4^+^ T cells that carry at least partially dysfunctional CCR5 co-receptors. These genetically modified cells are less susceptible to HIV-1 infection^6^. Recently Tebas *et al.*^1^ used zinc finger based gene editing targeting CCR5 gene and modified a fraction (11–28%) of host CD4^+^ T cells^1^. They found that genetically modified T cells have a survival advantage over unmodified T cells upon ART interruption and that gene therapy can boost total CD4^+^ T cell count. However, their data suggest that gene therapy does not suppress the viral load (see Supplementary Table S1) possibly because the genetically modified T cells were still susceptible to HIV-1 infection. Other researchers are targeting the virus by knocking down CCR5 using si/shRNA constructs or by inhibiting viral fusion (using C46 peptides)^3^,^7^,^8^,^9^. In summary, most of the current gene therapy trials try to decrease the infection rate by targeting viral entry via the CCR5 co-receptor^10^,^11^. Because targeting viral entry reduces the susceptibility of a subset of the target CD4^+^ T cells, CD4^+^ T cell counts should increase and the viral load should decrease.

Since the data remain inconclusive (Supplementary Table S1), we ask the question: is CCR5 gene therapy expected to reduce the viral load over the long term, and to replace lifelong ART? Using several generic mathematical models we show that the viral load will not necessarily decrease, and propose to include a suicide gene in the genetically modified T cells to develop a much more efficient therapy.

## Results and Discussion

### Typical HIV infection

**Figure 1:**
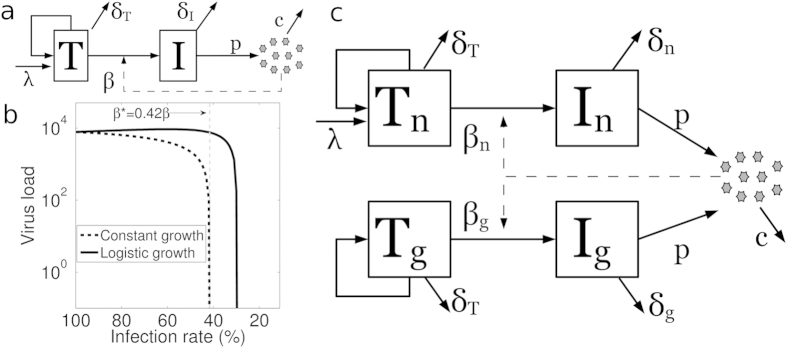
Models of the HIV infection. (**a**) Schematic of the *typical HIV model*. The uninfected CD4^+^ T cells can self-renew (logistic growth), die at rate *δ*_*T*_ day^−1^, and get infected at rate *β* particle^−1^ day^−1^. To model the influx from the naive compartment, uninfected T cells additionally get replenished at rate *λ* cells day^−1^. Infected cells produce *p* virus particles cell^−1^ day^−1^ and die at rate *δ*_*I*_ day^−1^. Viruses are cleared at rate *c* day^−1^ (see section Models). (**b**) The infection rate in the *typical HIV model* (*β*) was reduced, and is expressed as a percentage of the initial infection rate (*β*_0_). Only once the infection rate is reduced below a certain threshold (*β* < β^*^; vertical gray dashed line) will the viral load start to decrease. Several HIV-1 models let uninfected T cells be replenished at a constant rate from thymus, bone marrow, naive, and memory T cells^13^,^14^,^23^. A quantitavily similar model with a constant rate of replenishment (i.e. *λ* = 14.3 and *r* = 0 in Eq. 1) also has the property that *β* has to be decreased markedly (here more than 50%) to have a significant effect on the viral load^14^ (black dashed line). Modeling one *μ*l of blood, the following parameter values were used: *r* = 0.06 day^−1^, *K* = 1500 cells, *δ*_*T*_ = 0.02 day^−1^, *β*_0_ = 3.6 × 10^−6^ particle^−1^ day^−1^, *δ*_*I*_ = 1 day^−1^, *p* = 2.14 × 10^4^ virus particles cell^−1^ day^−1^, *c* = 23 day^−1^ (see section Models). The values of *λ, r*, and *K* were chosen to have 1000 T cells per *μl* blood in the virus-free steady state. (**c**) For the *GT model*, we model two populations of uninfected T cells (normal and genetically modified) sharing logistic growth. Normal uninfected T cells additionally get replenished at rate *λ* cells day^−1^. Normal and genetically modified T cells get infected at rate *β*_*n*_ and *β*_*g*_ particle^−1^ day^−1^ (where *β*_*g*_ ≤ *β*_*n*_), respectively. Infected cells die at rate *δ*_*n*_ and *δ*_*g*_ day^−1^.

To model HIV-1 infection, we considered that the target uninfected T cells are largely self-renewing and model this with a logistic growth for uninfected dividing *T*_*EM*_ cells, with a small constant influx from naive T cells becoming effector/memory cells^2^,^12^,^13^. Uninfected T cells die at rate *δ*_*T*_ and become infected at rate *β*. Infected T cells (*I*) die at rate *δ*_*I*_ producing virus particles (*V*) at rate *p* per infected cell; viruses are cleared at rate *c* (Fig. 1a and Eqs 1, 2, 3). In short, we wrote a *typical model* for the main features of HIV-1 infection^13^,^14^,^15^.


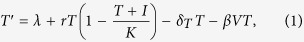



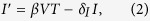



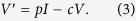


To derive analytical solution for the steady state, we make the reasonable assumption that the uninfected T cell population is largely self-renewing (i.e. *λ* = 0 in Eq. 1)^2^,^12^ to obtain the following steady state:


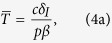



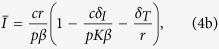



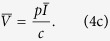


Our *typical HIV model* shows that decreasing the infection rate (*β*) increases the uninfected T cell count 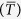 (Eq. 4a; Supplementary Fig. S1). However, decreasing the infection rate (*β*) can either increase or decrease the viral load (decreasing *β* increases the first term *cr*/*pβ* and decreases the term in the parentheses for Eqs 4b and 4c). Thus, if the infection rate does not decrease substantially gene therapy can actually have either no effect or even marginally increase the viral load (note the slight increase in the viral load for *β* ≥ β^*^; Fig. 1b). Obviously decreasing the infection rate markedly (*β* < β^*^; i.e. a nearly complete suppression of CCR5) should always decrease the viral load (Fig. 1b) as the infection can no longer sustain itself^14^. Note that we assumed *λ* = 0 in Eq. 1 to present a simple analytical solution, and that similar results are obtained when *λ* > 0 (Eq. 5 and Supplementary Fig. S4a). For all subsequent analysis, we use the full model with *λ* > 0.

### Inefficient HIV-1 gene therapy

Current gene therapies generate modified T cells with either homozygous or heterozygous disruption of the CCR5 gene to reduce the expression of the CCR5 co-receptor on host T cells^1^,^6^. However, genetically modified T cells remain susceptible to infection by HIV-1 unless both alleles of CCR5 gene are completely disrupted^3^. In the example of Fig. 1b we find that the infection rate has to be reduced by more than 58% to reduce the viral load. Given that gene therapy cannot ensure complete disruption of the CCR5 gene, it is unclear if this substantial decrease in the infection rate is achieved by current gene therapies.

**Figure 2:**
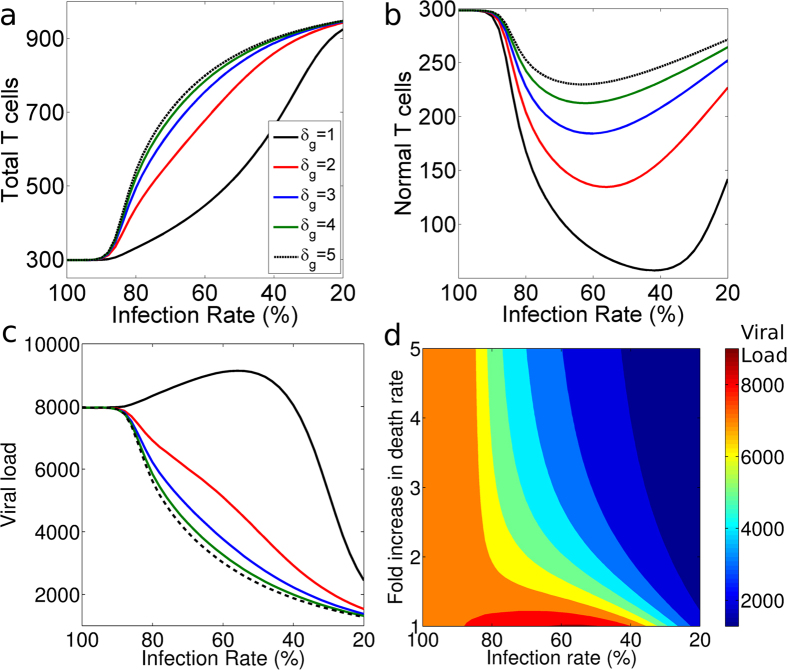
The effects of gene therapy (i.e. decreasing the infection rate *β*_*g*_) and the introduction of suicide gene (i.e. increasing the death rate, *δ*_*g*_, of the genetically modified T cells) on the steady state T cell count and the steady state viral load. We kept the infection rate of unmodified target cells constant (i.e. *β*_*n*_ = 3.6 × 10^−6^) and varied the infection rate of the genetically modified cells (*β*_*g*_). The value of *β*_*g*_ is given as a percentage of the *β*_*n*_ value, where 100% means that *β*_*g*_ = *β*_*n*_ and 20% means that *β*_*g*_ = 0.2*β*_*n*_. The effect of a change in the death rate of genetically modified cells (*δ*_*g*_) is shown by different lines. Black lines represent the current CCR5 gene therapies (with *δ*_*g*_ = *δ*_*n*_). The value for *δ*_*g*_ was changed as a fold increase of *δ*_*n*_. (**a**) Decreasing the infection rate (*β*_*g*_) of genetically modified cells increases the total T cell count (black line). (**b**) Decreasing the infection rate (*β*_*g*_) of genetically modified cells decreases the normal uninfected T cell count (black line). Decreasing the infection rate (*β*_*g*_) below a threshold rescues the normal uninfected T cell count. (**c**) Decreasing the infection rate (*β*_*g*_) of genetically modified cells can increase the viral load slightly for a mildly effective gene therapy. A suicide gene (to increase *δ*_*g*_) expressed in infected genetically modified cells increases the total T cell count (**a**), rescues the normal uninfected T cell count (**b**), and decreases the viral load (**c**) indicated by different colors (see legend in Fig. 2a). To initialize the *GT model*, we replaced 10% of the *T*_*n*_ cells by *T*_*g*_ cells in the infected steady state and we ran the model until the steady state is approached. Parameters: thymic influx for *T*_*n*_ (*λ*) = 1 cells *μl*^−1^ day^−1^, logistic growth parameter for *T*_*n*_ and *T*_*g*_ (*r*) = 0.057 day^−1^, and death rate of *I*_*g*_ (*δ*_*g*_); the other parameters remain the same (Fig. 1). The values for *r* and *λ* were chosen to have *T*_*n*_ = 1000 cells per *μl* blood as virus-free steady state using parameter values given in^22^,^23^,^24^. (**d**) The effect of variation of *β*_*g*_ along with *δ*_*g*_ in *GT model* (see section Models). The colors indicate the steady state viral load.

To study the efficacy of gene therapy, we make a gene therapy (GT) model from the typical model by allowing for two T cell populations: normal host T cells (*T*_*n*_) and genetically modified T cells (*T*_*g*_) (described in Fig. 1c and section Models). The *GT model* suggests that CCR5 gene therapy should always increase the total T cell count (*T*_*n*_ + *T*_*g*_: Fig. 2a) but will always decrease the normal T cell count (*T*_*n*_: Fig. 2b). The competition between the normal T cells and the genetically modified T cells can thus reduce the diversity of the T cell repertoire, as there will be fewer normal T cells.

The *GT model* suggests that the viral load is only guaranteed to decrease if the infection rate of the genetically modified T cells falls below a certain threshold i.e., less than 40% of that of the normal T cells (*β*_*g*_ < 0.4*β*_*n*_; see black line in Fig. 2c). Similar to the results shown in Fig. 1b, gene therapy does not decrease the viral load (see Fig. 2c,d, 0.4*β*_*n*_ ≤ *β*_*g*_) if the infection rate of the genetically modified T cells does not decrease substantially.

Gene therapy cannot guarantee homozygous CCR5 gene disruption since the majority of the modified T cells will be heterozygous for the CCR5 gene^3^. The infection rate of CD4^+^ T cells from individuals genetically heterozygous for the CCR5Δ-32 mutation (where all cells have the CCR5 disruption) is reduced by a maximum of 65%, making them slow progressors^3^. Unlike the individuals carrying the CCR5Δ-32 mutation, gene therapy can modify only a fraction of T cells (11%–28%) most of which will still be susceptible to HIV-1 infection (because they will be heterozygous for the CCR5 gene). Since only a fraction of T cells is modified, current gene therapies are not expected to decrease the viral load. For current CCR5 gene therapies to be successful and to control viral transmission, the infection rate has to be strongly reduced, which can only be achieved if almost all cells that are genetically modified become homozygous for the CCR5 deletion. This perfectly explains why introducing genetically modified T cells^1^ failed to decrease the viral load in the absence of ART, except in one patient who was heterozygous for the CCR5Δ-32 mutation (see Supplementary Table S1).

### Modifications to current gene therapy

Novel gene therapies targeting two or more steps in the HIV-1 life cycle have been proposed, and some are currently being tried^10^. Since we find that targeting CCR5 alone probably cannot replace ART, we studied whether or not combining CCR5 gene disruption with other factors could result in a more effective gene therapy. These other factors are: reducing the burst size (*p*) or increasing the virus clearance rate (*c*). Since these have the same effect as reducing the infection rate (*β*), they also result in an ineffective therapy (Supplementary Fig. S2).

#### HIV-1 suicide gene therapy

Since the uninfected T cell count increases linearly with the death rate, *δ*_*I*_, of the infected cells (Eq. 4a) but the viral load decreases with *δ*_*I*_ (Eqs 4b, 4c, 5b, and 5c), we quantified the effect of increasing the death rate of the infected cells. In practice, the death rate of the infected genetically modified T cells can be increased by introducing a suicide gene. Introducing a suicide gene and thus increasing the death rate of the infected genetically modified T cells (*δ*_*g*_) without CCR5 gene therapy (*β*_*g*_ = *β*_*n*_) does not affect the viral load as the genetically modified T cells without CCR5 disruption go extinct (Fig. 2a).

Remarkably, introducing a suicide gene with CCR5 gene therapy strongly suppresses the viral load (Fig. 2c). For example, a 50% decrease in the infection rate of the genetically modified T cells (*β*_*g*_) along with a 100% increase in the death rate of infected genetically modified T cells reduces the viral load by 50%. A 50% decrease in the infection rate (*β*_*g*_) without changing the death rate (i.e. without a suicide gene) does not decrease the viral load (it even slightly increased the viral load in Fig. 2c). Moreover, introducing a suicide gene in CCR5-disrupted genetically modified T cells increases the total T cell count (Fig. 2a) and rescues the normal T cell count (Fig. 2b). Thus the expression of a suicide gene in genetically modified T cells upon infection can markedly increase the effectiveness of CCR5 gene therapies (Fig. 2c). Moreover, HIV-1 has high mutation rates^16^ and can adapt alternate mechanisms (like receptor switching) to infect the genetically modified T cells. Introduction of suicide gene in CCR5 modified cells will provide protection against HIV-1 variants that evolve usage of alternate co-receptors because HIV-1 variants which infect using alternate receptors will activate the suicide gene in modified T cells.

The *GT model* was designed as a simple model of HIV-1 dynamics to help us gain insight into various features of CCR5 gene therapy^17^. To test its robustness, we studied several extensions allowing: for latent cells (Supplementary Fig. S3a); for cytotoxic T cell (CTL) responses (Supplementary Fig. S3b); for genetic modification of hematopoietic stem cells (Supplementary Fig. S3c and d); and for different stages of infected cells (i.e. an eclipse phase; Supplementary Fig. S3e). Irrespective of the model used, we predict that incomplete gene therapy targeting only the CCR5 receptor fails to reduce the viral load. However, a concomitant increase in the death rate of infected cells always resulted in an effective therapy.

In the *GT Model*, we considered that growth of target uninfected T cells is largely via self-renewal of *T*_*EM*_ cells with a small influx from the source (i.e. naive T cells becoming effector/memory cells). To test how this affects our results, we performed a parameter sensitivity analysis, and found that when contribution from the source is less than 10%, genetically modified (*T*_*g*_) cells can survive and form a major fraction of the T cell population. Hence the introduction of a suicide gene along with CCR5 gene therapy can be beneficial only when the contribution from the source is less than 10% (Supplementary Fig. S4a and b). When the contribution from the source is high, *T*_*g*_ cells either go extinct or are present in such low numbers that any form of CCR5 based gene therapy will not be effective. Since the CCR5 receptor is found on *T*_*EM*_ cells that are formed largely via self-renewal the contribution from the source should be low. Moreover, Tebas *et al.*^1^ found that the genetically modified cells can survive for long time, indicating that the source contribution is low. A similar parameter sensitivity analysis for the rate of infection (*β*_*n*_) of normal T cells confirmed that the introduction of suicide gene always reduces the viral load and increases the T cell count compared to the CCR5 gene therapy without suicide gene (Supplementary Fig. S4c and d).

## Conclusions

A great deal of effort is being made to improve HIV-1 gene therapy after seeing the “curative” effects of the CCR5Δ-32 mutation in the “Berlin patient”. Mitsuyasu *et al.*^8^ and Tebas *et al.*^1^ achieved an increase in CD4^+^ T cell counts within patients as predicted by Fig. 2a and Supplementary Fig. S1; however, neither gene therapy significantly reduced the viral load. Different suicide genes (e.g., HSV1-TK and iCasp9) are known to cause cell death when expressed at high concentration^18^. HIV-1 infected cells can be killed in a specific manner if a suicide gene (HSV1-TK or iCasp9) is successfully incorporated with the HIV-1 promoter and transactivation response element sequences^18^,^19^,^20^. Our models show that the therapeutic effect of targeting viral entry is markedly increased when the infected modified T cells undergo rapid cell death when they are infected. It therefore seems feasible and promising to combine a suicide gene with zinc finger or si/shRNA based constructs to knock down CCR5.

## Methods

### Mathematical models for HIV-1 gene therapy

We constructed a suite of models, considering various extensions like latency, cytotoxic T cell (CTL) responses, different stages of infected cells, to test the predictions of our simple model. We found that in all models CCR5 gene therapy is predicted to fail unless it is combined with a suicide gene. Mathematical models were simulated in MATLAB R2014b (http://www.mathworks.com/products/matlab/).

For the *typical HIV model*, we considered that *T* cells get replenished via logistic growth (dividing *T*_*EM*_ cells) and have a small contribution from another compartment (e.g. naive T cells). We consider that upon infection both uninfected (*T*) and infected T cells (*I*) compete in the logistic growth term (Eqs 1, 2, 3 and Fig. 1a). To obtain the simple steady state expression presented in the text (Eq. 4), we assumed that 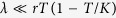 and 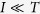. If we relax these assumptions the results do not change qualitatively as shown by the steady state expression obtained for the full *typical HIV model*:


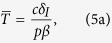



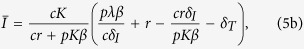



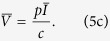


The *GT model* considers two populations of uninfected and infected T cells: normal uninfected (*T*_*n*_) and infected (*I*_*n*_) T cells, and genetically modified uninfected (*T*_*g*_) and infected (*I*_*g*_) T cells. *T*_*n*_ cells get replenished via logistic growth and have a small contribution from another compartment (e.g. naive T cells). We allowed all actively dividing cells (*T*_*n*_, *T*_*g*_, *I*_*n*_, and *I*_*g*_) to compete in the logistic growth of *T*_*n*_ and *T*_*g*_ cells. The virus particles (*V*) are produced from *I*_*n*_ and *I*_*g*_ (Eqs 6, 7, 8, 9, 10 and Fig. 1b). Parameters are described in the legend of Fig. 2:


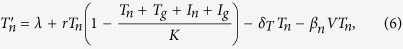



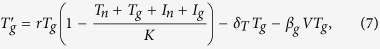



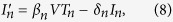



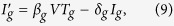



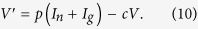


In the *Latent model*, we changed the dynamics for the infected cells by adding compartments for latently infected T cells (normal: *L*_*n*_ and genetically modified: *L*_*g*_). The equations for *T*_*n*_ cells, *T*_*g*_ cells, and *V* (Eqs 6, 7, and 10) remain the same, and the equations for infected cells are replaced by Eqs 11, 12, 13, 14. We added the following parameters: *f* = 0.995, *δ*_*L*_ = 0.0001, and *α* = 0.1, the other parameters remained the same:


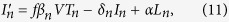



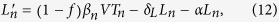



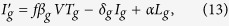



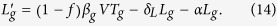


To model the effect of CTL killing, we consider that a fraction of infected T cells are killed by CTLs (Eqs 15 and 16, where *δ*_*E*_ = 0.9, *δ*_*n*_ = 0.1, *δ*_*g*_ = *d* × *δ*_*n*_) keeping equations 6, 7, and 10, and the remaining parameters the same (Fig. 2):


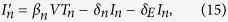



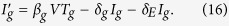


If CTLs are largely responsible for killing the infected T cells (*δ*_*E*_ = 0.9 and *δ*_*n*_ = 0.1), a suicide gene in *I*_*g*_ cells should kill the infected cells much more rapidly to curb HIV transmission (Supplementary Fig. S3b). This is not unreasonable because Marin *et al.*^18^ showed that suicide genes can indeed cause rapid cell death. Adding a CTL population with a competitive saturating function for CTL growth, and a mass-action term for CTL-mediated killing (Eqs 17, 18, 19) enables that the viral load depends on many parameters of the model^21^. Considering such a CTL model does not change the results qualitatively (data not shown):


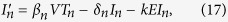



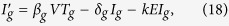



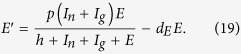


To study hematopoietic stem cell (HSC) transplantation as an alternative to conventional gene therapy with mature T cells, we allow HSC cells to contribute to the replenishment of genetically modified T cells. We replaced the *T*_*g*_ growth dynamics by equation 20 keeping Eqs 6, 8, 9, 10 and other parameters the same:


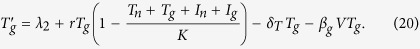


Althaus *et al.*^22^ recently described a within-host HIV model considering different sub-classes of HIV-1 infected cells, namely, latently infected cells (*L*1 and *L*2), persistently infected cells (*M*1 and *M*2), defectively infected cells (*D*), newly infected cells (*I*1; without integrated virus), infected cells with integrated proviral DNA (*I*2), infected cells with increasing HIV transcriptional activity (*I*3, *I*4, and *I*5) and virus producing cells (*I*6). The *I*1, *I*2, *I*3, *I*4, and *I*5 sub-classes of HIV-1 infected cells describe the intracellular eclipse phase of HIV-1 dynamics. We extended this model to allow for a population of normal T cells (*i* = 1) and genetically modified T cells (*i* = 2) (Eqs 21, 22, 23, 24, 25, 26, 27, 28, 29, 30, 31, 32, 33), and extend the model to have logistic growth for *T*_1_ and *T*_2_ cells:


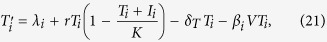



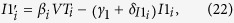



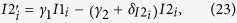



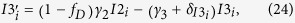



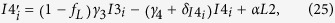



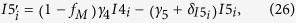



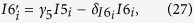



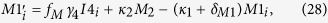



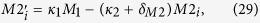



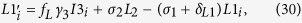



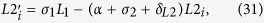



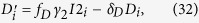



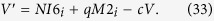


The following parameters were kept the same^22^: *δ*_*T*_ = 0.0165, *β*_1_ = 1.35 × 10^−6^, 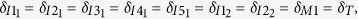

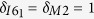
, *δ*_*D*_ = 8.3 × 10^−5^, *δ*_*L*1_ = *δ*_*L*2_ = *δ*_*D*_, *f*_*D*_ = 0.141, *f*_*L*_ = 0.0035, *f*_*M*_ = 0.25, *γ*_1_ = 3, *γ*_2_ = 6, *γ*_3_ = *γ*_4_ = *γ*_5_ = 12, *σ*_1_ = 0.0789, *σ*_2_ = 1, *κ*_1_ = 0.103, *κ*_2_ = 1, *α* = 2.67 × 10^−3^, *N* = 2.14 × 10^4^, and *c* = 23. We added the following parameters to make the virus-free steady state equivalent to that of the Althaus^22^ model: *λ*_1_ = 1, *λ*_2_ = 0, *r* = 0.0856, and *K* = 5000. To model the expression of suicide genes, we increased the death rate of genetically modified infected cells expressing viral RNA to 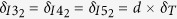, and 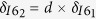.

## Additional Information

**How to cite this article**: Pandit, A. and de Boer, R. J. HIV-1 CCR5 gene therapy will fail unless it is combined with a suicide gene. *Sci. Rep.* **5**, 18088; doi: 10.1038/srep18088 (2015).

## Supplementary Material

Supplementary Information
