# Supplementary material for: HIV-1 CCR5 gene therapy will fail unless it is combined with a suicide gene
Source: Sci Rep. 2015 Dec 17;5:18088. doi: 10.1038/srep18088 (PMC4682191; doi:10.1038/srep18088)
Supplement: Supplementary Information [file srep18088-s1.pdf]

HIV-1 CCR5 gene therapy will fail unless it is combined with a suicide gene

Aridaman Pandit[1] and Rob J de Boer[1]
[1]Theoretical Biology and Bioinformatics, Utrecht University, Utrecht, The Netherlands

Supplementary Information

Table S1. A summary of the viral load observed in the study of Tebas et al.[1]

Patient	Set-point VL ($\log_{10}$ copies/ml)	VL after gene therapy ($\log_{10}$ copies/ml)	Comments
201	3.27	3.99	Moderate increase
203	4.08	3.92	Mild decrease
204	4.73	4.78	ART re-initiated because of high VL
205	5.21	1.53	Individual was heterozygous for CCR5Δ32
251	4.72	5.22	ART re-initiated because of high VL
253	4.16	4.11	Mild decrease

The patients shown here underwent a 12-week interruption to ART, 4 weeks after receiving the gene therapy. A marked decline of the viral load was only observed in patient 205, who was later discovered to be heterozygous for CCR5Δ32.[1] Thus, the gene therapy potentially resulted in homozygous CCR5 disruption in modified CD4+ T cells, indicating that complete disruption of CCR5 gene can drastically decrease the viral load. In the other five patients the viral load went up (3/5) or mildly down (2/5). The viral load values were obtained using WebPlotDigitizer (http://arohatgi.info/WebPlotDigitizer) from data given in Tebas et al.[1]

References

1. Tebas, P. *et al.* Gene editing of CCR5 in autologous CD4 T cells of persons infected with HIV. *New England Journal of Medicine* **370**, 901–910 (2014).

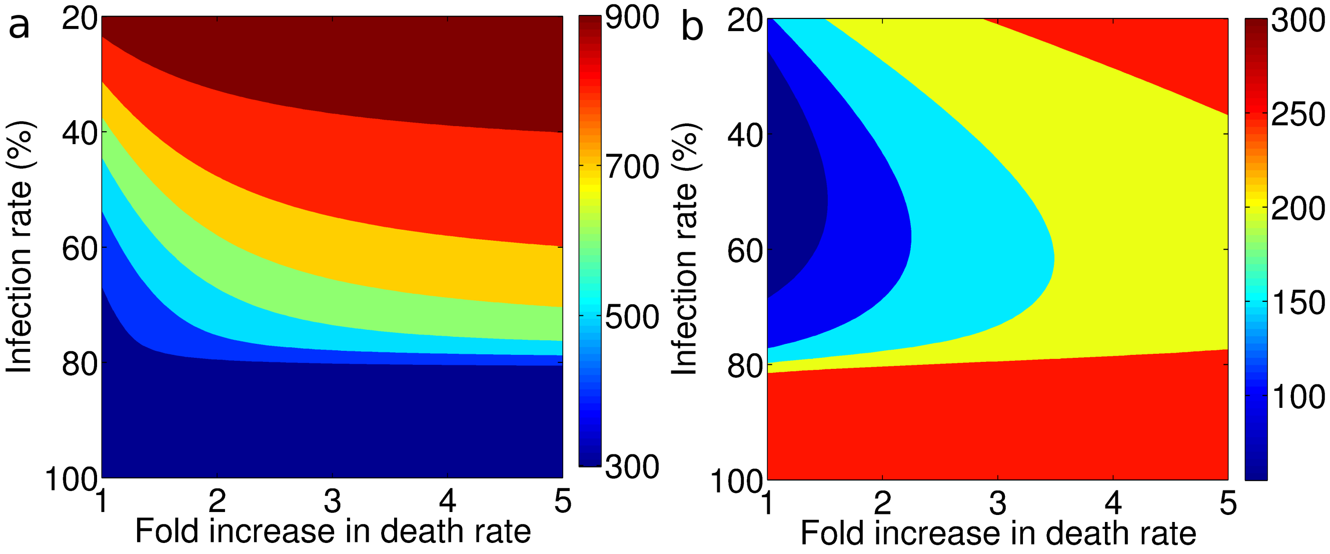

Figure S1. The effect of decreasing the infection rate (β_g) and increasing the death rate (δ_g) of genetically modified cells on the steady state: (a) total CD4+ T cell count ($\overline{T_n} + \overline{T_g}$), and (b) normal CD4+ T cell count ($\overline{T_n}$). The colors indicate steady state T cell counts, (a) $\overline{T_n} + \overline{T_g}$ or (b) $\overline{T_n}$, for the same parameter values as considered in Figure 2a. A decrease in β_g can decrease the counts and hence the diversity of the normal T cells, T_n (Panel b). A concomitant increase in δ_g retains the counts (and hence the diversity) of the T_n cells much better (Panel b).

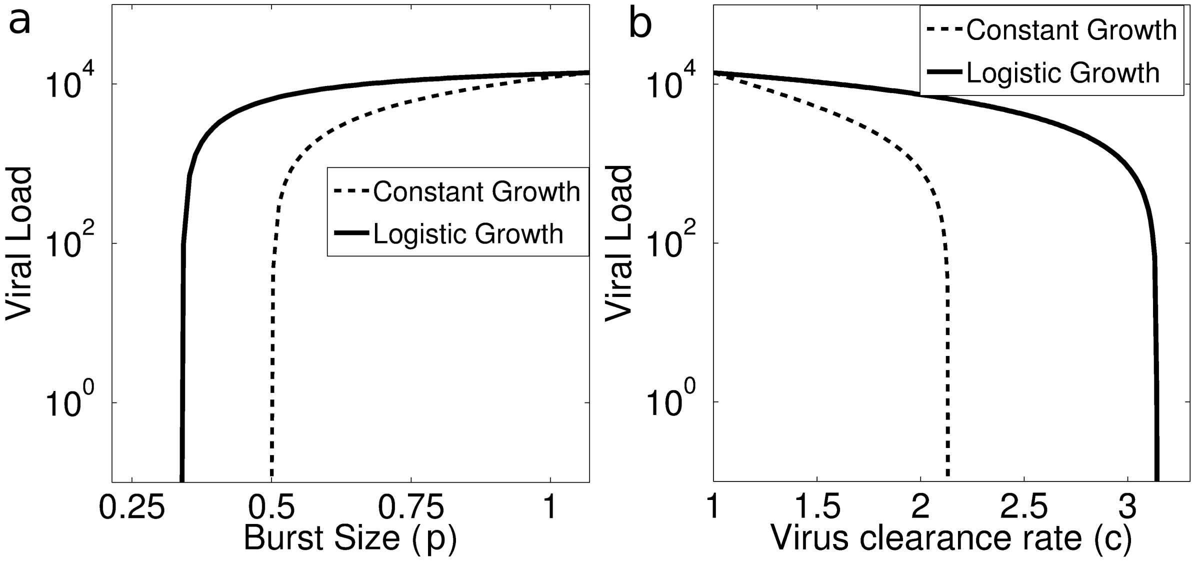

Figure S2. The effect of changing (a) the burst size, p, and (b) the virus clearance rate, c, on viral load in the typical HIV model (Eqs. 1-3).

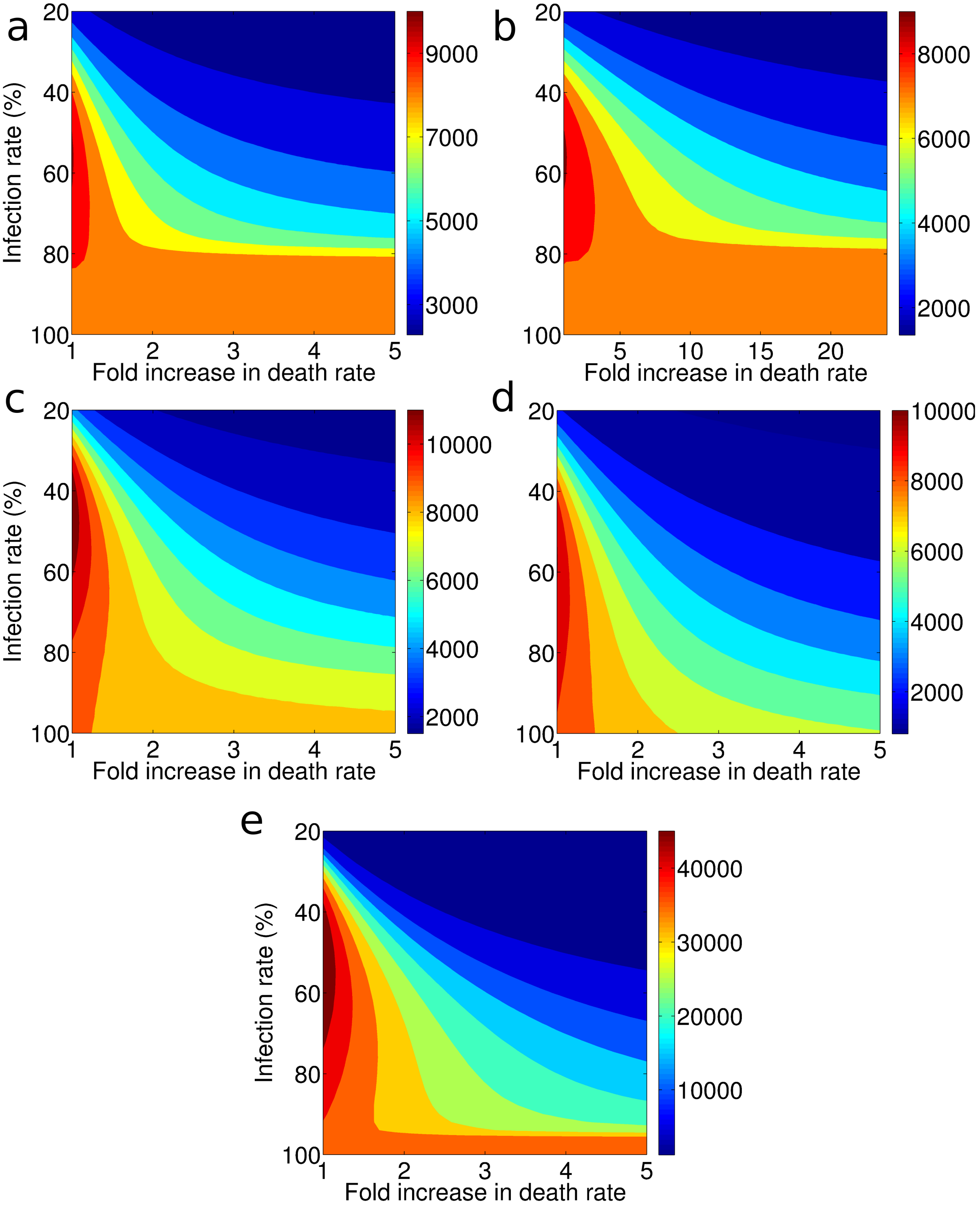

Figure S3. The effect of decreasing the infection rate (β_g) and increasing the death rate (δ_g) of genetically modified cells in: (a) the *Latent model* (Eqs. 6, 7, 10-14); (b) the *CTL model* (Eqs. 6, 7, 10, 15, and 16); (c) the model considering 25% hematopoietic stem cells to be genetically modified ($\lambda_2 = 0.33\lambda_1$); (d) the model considering 50% hematopoietic stem cells to be genetically modified ($\lambda_2 = \lambda_1$ in Eqs. 6, 8-10, and 20); and (e) the model considering different stages of infected cells described in Methods (Eqs. 21-33).

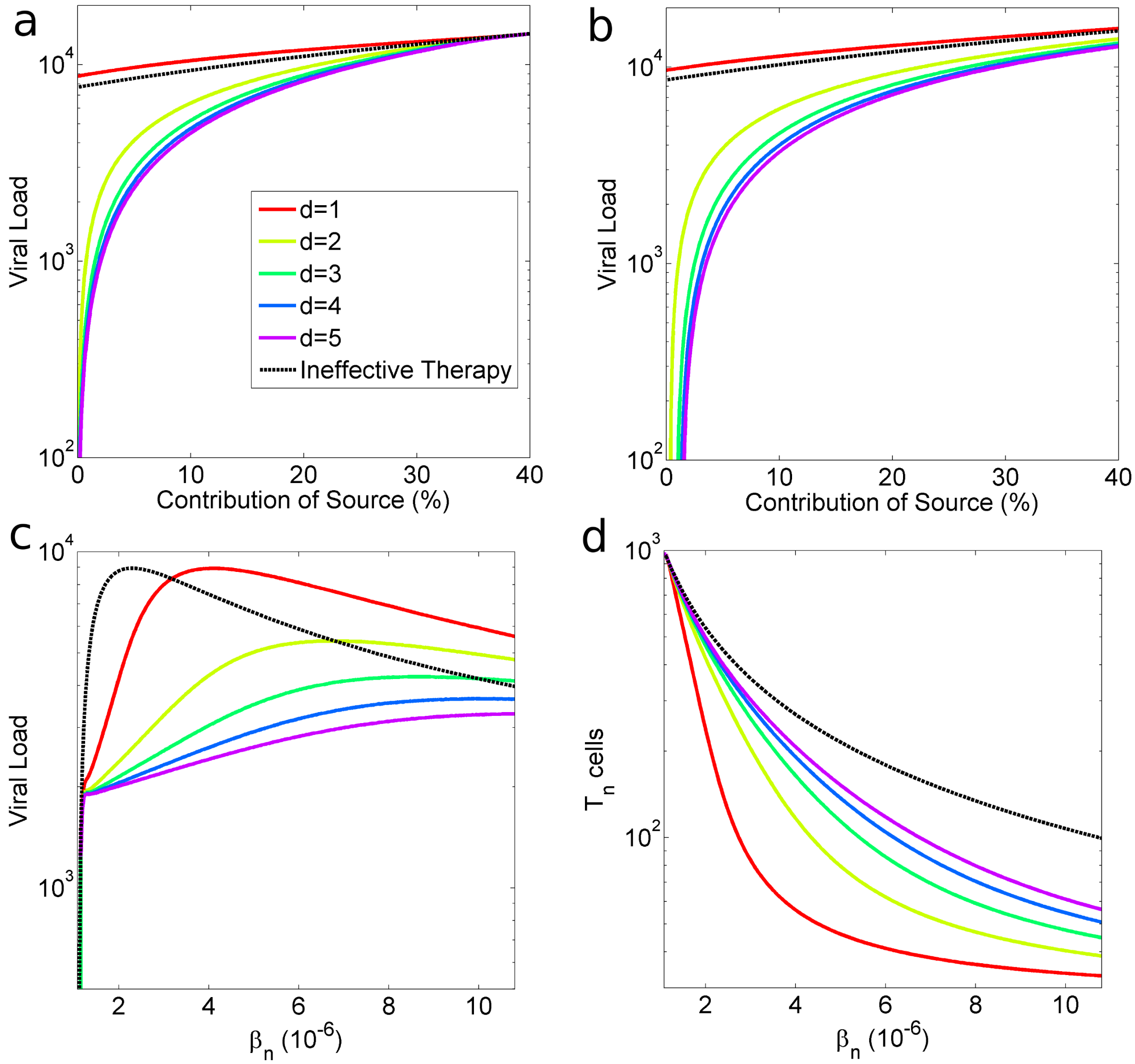

Figure S4. Parameter sensitivity analysis. In Figure 2, we considered the case where 5% of the normal uninfected T cells (T_n) come from the source (naive T cells) and 95% of the T_n cells are formed via self-renewal (i.e. $\lambda = 1$ cells μl^{-1} day^{-1}, $r = 0.057$ and $K = 1500$ in Eq. 1). Here we study the effect of the contribution from the source on a CCR5 suicide gene therapy. We performed a parameter sensitivity analysis considering $r = 0.06$ and $\overline{T_n} = 1000$ (Fig. 1) for the virus free steady state, and varied λ and K solving K from $K = r\overline{T_n}^2/(\lambda + (r - \delta_T)\overline{T_n})$ (derived from Eq. 1). In panel (a) we show the effect of the relative contribution from the source for T_n cells with a heterozygous CCR5 disruption (i.e. $\beta_g = 0.5\beta_n$) considering normal gene therapy i.e. 0% contribution from the source for T_g cells. In panel (b) we repeat the analysis considering a stem cell based gene therapy i.e. 5% contribution from the source for T_g cells. In panels (c) and (d) we perform a similar parameter sensitivity analysis by varying the rate of infection of the unmodified T cell β_n, setting other parameters the same as Fig. 2. In panel (c) we show that for large range of β_n values a suicide gene always results in a reduction of the virus load compared to the current CCR5 gene therapy (red line). In panel (d) we show that an increase in β_n results in a decrease of the T_n cells. Since we are simulating the chronic stage of HIV-1 infected individuals, we consider normal T cell counts of about 300 cells μl^{-1}. For high rates of infection ($\beta_n \geq 5.4 \times 10^{-6}$), T_n is less than 200 cells μl^{-1} which is too low for a normal chronic stage, as one expects AIDS to develop. Nevertheless, a suicide gene would always improve the normal T cell count compared to the current CCR5 gene therapy (red line).
